# Quantifying the Diamagnetic and Paramagnetic Components of Beta‐Amyloid Plaques Using Decomposed Quantitative Susceptibility Mapping in Mouse Models of Alzheimer's Disease

**DOI:** 10.1002/nbm.70383

**Published:** 2026-09-01

**Authors:** Juan Liu, Jingjia Chen, Season K. Wyatt‐Johnson, Jie Chen, Zhuoheng Liu, Hongbo Wu, Li Feng, Randy R. Brutkiewicz, Nian Wang

**Affiliations:** ^1^ The University of Texas Southwestern Medical Center Dallas Texas United States; ^2^ Bernard and Irene Schwartz Center for Biomedical Imaging, Department of Radiology New York University Grossman School of Medicine New York New York United States; ^3^ Center for Advanced Imaging Innovation and Research (CAI2R), Department of Radiology New York University Grossman School of Medicine New York New York United States; ^4^ Department of Microbiology and Immunology Indiana University School of Medicine Indianapolis Indiana United States; ^5^ Department of Biomedical Engineering The University of Texas Southwestern Medical Center Dallas Texas United States; ^6^ Peter O'Donnell Jr. Brain Institute The University of Texas Southwestern Medical Center Dallas Texas United States

**Keywords:** Alzheimer's disease, beta‐amyloid plaque, diamagnetic, high‐resolution, paramagnetic, QSM

## Abstract

High‐resolution quantitative susceptibility mapping (QSM) combined with susceptibility source decomposition provides a powerful approach for investigating the magnetic susceptibility alterations in Alzheimer's disease (AD). In this study, ex vivo three‐dimensional multi‐echo gradient‐echo (mGRE) images of 5xFAD and $App^{\text{SAA}}$ mouse brains were acquired at 30 μm isotropic resolution using a 9.4 T MRI scanner. QSM was reconstructed and decomposed into diamagnetic component susceptibility (DCS) and paramagnetic component susceptibility (PCS). Individual amyloid‐beta (Aβ) plaques were automatically detected and their susceptibility properties were quantitatively characterized. DCS and PCS revealed subvoxel mixtures of diamagnetic and paramagnetic components. Compared with conventional QSM, DCS exhibited clearer plaque visualization and higher plaque detection sensitivity. Histological analysis revealed the coexistence of Aβ aggregates and ferritin in both mouse models, with most plaques measuring less than 70 μm in diameter. To assess the impact of spatial resolution on plaque visualization and susceptibility quantification, the acquired k‐space data were downsampled to isotropic resolutions of 45, 60, and 90 μm. As spatial resolution became coarser, plaque visibility degraded progressively, resulting in reductions in both the number of detectable plaques and the estimated plaque burden. Whole‐cortex plaque loading decreased from 8.9% at 30 μm to 3.1% at 90 μm in 5xFAD and from 6.5% to 2.6% in $App^{\text{SAA}}$ mice. In both models, detected plaques exhibited predominantly diamagnetic susceptibility, with the diamagnetic component accounting for approximately 80% of the total absolute susceptibility. These findings demonstrate that high‐resolution susceptibility mapping combined with source decomposition enables plaque‐level characterization of diamagnetic and paramagnetic contributions and provides complementary biomarkers for assessing Aβ pathology and associated iron dysregulation in preclinical mouse models of AD.

## Graphical abstract

Ex vivo MRI was performed on AD mouse models using high‐resolution multi‐echo gradient‐echo imaging on a 9.4T scanner to generate quantitative susceptibility maps (QSM) and separate diamagnetic and paramagnetic components. Amyloid‐β plaques were detected from the susceptibility maps. The plaques showed a dominant diamagnetic component associated with amyloid‐β and a paramagnetic contribution attributed to co‐localized iron deposition.

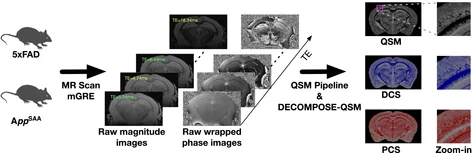

## 1 Introduction

Alzheimer's disease (AD) is a progressive neurodegenerative disorder that causes irreversible deterioration in memory and cognitive function, ultimately leading to dementia [1, 2]. Its main pathological features include the accumulation of amyloid‐beta (Aβ) plaques outside neurons [3, 4] and intracellular neurofibrillary tangles formed by abnormally phosphorylated tau protein [5], which together contribute to neuronal loss and brain atrophy. AD commonly affects the hippocampus and cortical regions, which are responsible for memory, learning, thinking, and reasoning. Although there is currently no cure for AD, accurate and early assessment of disease pathology is crucial for timely intervention, effective disease management, and improved patient care.

Quantitative susceptibility mapping (QSM) is a noninvasive magnetic resonance imaging (MRI) technique that quantifies spatial variations in tissue magnetic susceptibility [6, 7, 8]. Owing to its sensitivity to both paramagnetic and diamagnetic substances, QSM has been widely applied to the study of neurological disorders, including Parkinson's disease [9, 10, 11], multiple sclerosis [12, 13, 14], and AD [15, 16, 17]. In AD, Aβ aggregation, tau pathology, iron dysregulation, and alterations in myelin integrity can modify tissue magnetic susceptibility. Previous studies have reported associations between susceptibility alterations and major features of AD pathology, including amyloid burden, abnormal iron accumulation, and neurodegeneration, supporting the use of QSM as a complementary imaging approach for characterizing plaque‐associated tissue changes. Furthermore, QSM may complement molecular imaging modalities, including amyloid positron emission tomography (PET) [18, 19] and tau‐PET [20, 21], offering a non‐radioactive approach for investigating the interplay among iron homeostasis, amyloid pathology, and neurodegeneration.

Despite these strengths, QSM has important limitations as a biomarker for AD [22, 23]. Because QSM measures net magnetic susceptibility, it cannot distinguish the contributions from coexisting paramagnetic and diamagnetic sources. In AD, Aβ plaques are primarily diamagnetic because of their aggregated amyloid protein [17], whereas paramagnetic iron deposits frequently colocalize with Aβ aggregates [24, 25, 26]. These opposing susceptibility sources frequently colocalize within cortical and subcortical regions of brains affected by AD. When these sources are present in close spatial proximity, QSM cannot reliably resolve the individual contributions of diamagnetic and paramagnetic sources. As a result, QSM alone is insufficient to disentangle these distinct magnetic contributions, underscoring the need for susceptibility decomposition methods that can separately quantify diamagnetic and paramagnetic components and provide a more biologically specific characterization of plaque‐associated pathology.

To address this limitation, several methods have been developed to decompose total susceptibility into paramagnetic and diamagnetic components, including DECOMPOSE‐QSM [27], χ‐separation [28], $R_{2}^{*}$QSM [29], APART‐QSM [30], QSM‐ARCS [31], and χ‐sepnet [32]. These techniques can be broadly categorized into $R_{2}^{*}$‐based methods and $R_{2}^{\prime }$‐based methods [33]. The $R_{2}^{*}$‐based approaches rely solely on gradient‐echo (GRE) acquisitions to estimate susceptibility components, offering greater acquisition efficiency and simpler implementation. In contrast, $R_{2}^{\prime }$‐based methods require both GRE and spin‐echo (SE) data to explicitly quantify reversible dephasing effects, thus potentially providing greater accuracy in source separation [34, 35]. Susceptibility separation techniques have been applied to investigate a range of neurological conditions, including brain development and aging [36, 37, 38, 39], Parkinson's disease [40, 41], multiple sclerosis [34, 42, 43, 44], and AD [17, 45, 46, 47].

In the context of AD, susceptibility decomposition enables the independent assessment of paramagnetic susceptibility, which mainly reflects iron accumulation, and diamagnetic susceptibility, which is associated with substances such as Aβ or myelin. Using phantom experiments, Gong et al. [17] demonstrated that Aβ aggregates are intrinsically diamagnetic and showed that diamagnetic and paramagnetic susceptibility maps provide complementary contrast for identifying amyloid and iron deposition in mouse models of AD. Ahmed et al. [45] applied DECOMPOSE‐QSM to separate diamagnetic and paramagnetic susceptibility components in AD patients and healthy controls. They found significantly lower absolute DCS values in white matter and CSF, and demonstrated associations between DCS and tau‐PET in gray matter regions affected by tau deposition, suggesting that DCS can serve as a sensitive marker for tracking tau aggregation and demyelination in AD. Yao et al. [46] used APART‐QSM to simultaneously quantify Aβ and iron in postmortem human brains from individuals with AD and controls. They found that although Aβ and iron colocalize in the medial parietal and parahippocampal cortices, they also show distinct spatial distribution patterns. Joshi et al. [47] applied susceptibility decomposition to ex vivo brains of PS19‐P301S tauopathy mice and age‐matched wild‐type controls at 11.7 T and suggested that susceptibility source separation methods may provide sensitive biomarkers for detecting microstructural changes in tauopathies well before macroscopic structural pathology emerges. However, existing studies have been limited by insufficient spatial resolution, which prevents direct visualization of individual plaques and characterization of their susceptibility properties.

In this study, we investigated the magnetic susceptibility characteristics of amyloid plaques in two mouse models of AD using high‐resolution MRI combined with susceptibility decomposition. The two mouse models were 5xFAD and $App^{\text{SAA}}$ [48]. The 5xFAD model is an aggressive overexpression model characterized by rapid plaque accumulation and cognitive deficits. Dense‐core and diffuse plaques appear as early as two months of age and progress rapidly throughout cortical and subcortical regions [49]. In contrast, the $App^{\text{SAA}}$ model is a sophisticated genetic mouse model of AD amyloid pathology and uses a knock‐in approach rather than the traditional, less‐physiological overexpression of transgenes [50]. This model approaches amyloidogenesis and inflammation from a complementary perspective, emphasizing SAA's contribution to plaque formation and distinct inflammatory responses. Three‐dimensional multi‐echo gradient‐echo (mGRE) data were acquired at 30 μm isotropic resolution and used to reconstruct QSM. DECOMPOSE‐QSM [27] was then applied to generate diamagnetic component susceptibility (DCS) and paramagnetic component susceptibility (PCS) maps. Individual plaques were detected and their susceptibility properties were quantified. Histological validation was performed using immunofluorescence staining to visualize Aβ aggregates and ferritin deposits. We further examined the effect of spatial resolution on plaque detection and susceptibility quantification by downsampling the data from 30 μm to isotropic resolutions of 45, 60, and 90 μm. The objective of this study was to characterize plaque susceptibility properties and investigate how spatial resolution affects plaque detectability and susceptibility quantification in two AD mouse models.

## 2 Materials and Methods

### 2.1 MRI Data

All animal experiments were conducted in accordance with the guidelines approved by the local Institutional Animal Care and Use Committee (IACUC). Mice were deeply anesthetized and subsequently euthanized by transcardial perfusion fixation [51, 52]. Ex vivo MRI was performed on nine 5xFAD mice (12–18 months of age, all male) and seven $App^{\text{SAA}}$ mice (11–12 months of age, all male). All images were acquired using a 9.4 T Bruker BioSpec system (Bruker BioSpin, Ettlingen, Germany) equipped with a four‐channel cryoprobe RF coil. Two 3D mGRE acquisitions with monopolar readouts and different first‐echo times were acquired and interleaved to reduce the effective echo spacing, providing a better signal‐to‐noise ratio (SNR) in high‐field MRI because of fast T2^∗^ decay. The imaging parameters were as follows: matrix size = 400 × 400 × 240, field of view (FOV) = 12.0 × 12.0 × 7.2 mm^3^, spatial resolution = 30 × 30 × 30 μm^3^, flip angle = 50°, TE1/ΔTE/TE8 = 3.04/3.4/30.24 ms and 4.74/3.4/31.94 ms for the first and second acquisitions, respectively, repetition time (TR) = 200 ms, receiver bandwidth = 250 kHz, 1 average, partial Fourier acquisition. Each acquisition required approximately 4 h, resulting in a total acquisition time of approximately 8 h for each mouse.

### 2.2 Histological Data

After MRI acquisition, one scanned brain from a 5xFAD mouse and one from an $App^{\text{SAA}}$ mouse were cryoprotected in 15% sucrose overnight and subsequently transferred to 30% sucrose until fully equilibrated. Brains were then frozen on dry ice and stored at $-80^{\circ }$C until sectioned at 30 μm on a cryostat. Immunofluorescence staining on free‐floating tissue sections was performed as previously described [53]. Sections were washed in 1× PBS containing 0.1% Triton X‐100 (Sigma) for 5 minutes, followed by permeabilization in 1× PBS containing 3% Triton X‐100 for 30 min at room temperature (RT). Sections were then incubated with mouse Fc blocker (553242, BD Pharmingen, Plainfield, IN) for 1 h, followed by blocking in goat immunostaining buffer (5% goat serum [0060‐01, SouthernBiotech, Birmingham, AL], 0.3% BSA, 0.3% sodium azide, 1× PBS containing 0.3% Triton X‐100) for 4 h at RT. Sections were incubated with the following primary antibodies for 48 h on a rotating platform at $4^{\circ }$C: rabbit anti‐ferritin (polyclonal, 1:50, PA1‐29381, ThermoFisher Scientific, Waltham, MA) and mouse anti‐amyloid‐β (monoclonal, 1:50, NBP2‐23075, Novus Biologicals, Centennial, CO). Following primary antibody incubation, sections were washed three times in 1× PBS containing 0.1% Triton X‐100 and then incubated with secondary antibodies for 2 h at RT: goat anti‐rabbit IgG Alexa Fluor 568 (1:5000, A11011, ThermoFisher Scientific) and goat anti‐mouse IgG2b Alexa Fluor 488 (1:5000, A21241, ThermoFisher Scientific). Nuclei were counterstained with DAPI (62248, ThermoFisher Scientific). Following secondary antibody incubation, sections were washed three times in 1× PBS containing 0.1% Triton X‐100 and mounted on glass slides using Vectashield Antifade mounting medium (H‐1000, Vector Laboratories) and sealed with CoverGrip Coverslip Sealant (23005, Biotium, Fremont, CA). Fluorescence images were acquired using a Nikon Eclipse Ti inverted microscope equipped with Plan Fluor 10× /1.30 and 100× /1.49 objectives and NIS‐Elements software (Nikon Instruments).

We used ITK‐SNAP [54] to manually measure plaque diameters in the Aβ‐stained histological images. The resulting plaque size distributions were then visualized using histograms. In addition, we calculated plaque volumes from the measured diameters by assuming that the plaques were spherical.

### 2.3 QSM Processing

The image sets from the two mGRE acquisitions were coregistered using Advanced Normalization Tools (ANTs) [55] with a rigid transformation comprising rotation and translation and then combined across echoes by interleaving. A locally low‐rank (LLR) denoising algorithm [56, 57] was applied to the complex‐valued mGRE data to suppress noise while preserving fine anatomical structures. Brain masks were generated from the magnitude image using an in‐house deep learning method. Phase images from each echo were unwrapped using a combined spatiotemporal phase‐unwrapping approach [58]. Background field contributions were subsequently removed using the variable‐kernel sophisticated harmonic artifact reduction for phase data (V‐SHARP) [59], with a maximum spherical mean value (SMV) kernel radius of 600 μm, to obtain the local tissue‐induced magnetic field. Finally, the local field map at each echo time was independently processed using Nonlinear Dipole Inversion (NDI) [60] to generate per‐echo QSM maps.

**FIGURE 1:**
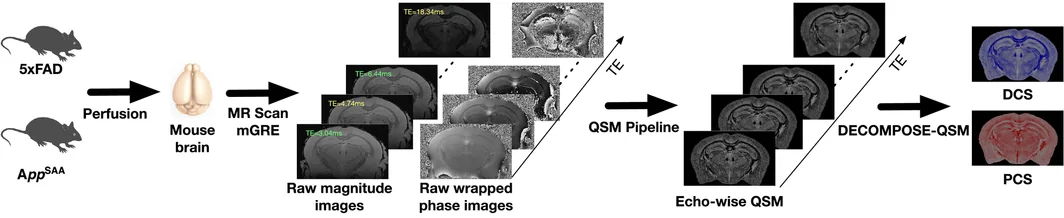
Schematic overview of the data acquisition and processing pipeline. The 5xFAD and $App^{\text{SAA}}$ mice were perfusion‐fixed, and their excised brains were scanned using a 9.4 T MRI system with three‐dimensional multi‐echo gradient‐echo acquisitions. The acquired data were reconstructed to generate magnitude and phase images. The phase images were then processed to generate per‐echo QSM, followed by susceptibility source separation using DECOMPOSE‐QSM to derive diamagnetic component source (DCS) and paramagnetic component source (PCS) maps.

The complete data‐processing pipeline is summarized in Figure 1.

### 2.4 Susceptibility Source Separation

Susceptibility decomposition is based on the principle that diamagnetic and paramagnetic susceptibility sources affect MRI signals differently through their contributions to frequency shift [61] and transverse relaxation rate [62]. When diamagnetic ($\chi_{-}$) and paramagnetic ($\chi_{+}$) sources coexist within a voxel, the observed frequency shift reflects the net susceptibility ($\chi_{-}+\chi_{+}$), whereas the transverse relaxation rate $R_{2}^{\prime }$ depends on the sum of their absolute susceptibility magnitudes ($\left|\chi_{-}|+|\chi_{+}\right|$) under the static dephasing regime. This biophysical relationship provides the theoretical foundation for susceptibility separation methods, which decompose the total magnetic susceptibility map into diamagnetic ($\chi_{-}$) and paramagnetic ($\chi_{+}$) component maps.

In this study, we used DECOMPOSE‐QSM to obtain PCS and DCS maps. DECOMPOSE‐QSM employs a biophysical multi‐compartment model that uses multi‐echo GRE data to disentangle the contributions of coexisting paramagnetic and diamagnetic susceptibility sources. The model represents the signal as arising from three compartments: a paramagnetic compartment with susceptibility $\chi_{+}$ and transverse relaxation rate $R_{2,+}^{*}$, a diamagnetic compartment with susceptibility $\chi_{-}$ and transverse relaxation rate $R_{2,-}^{*}$, and a reference compartment with susceptibility $\chi_{0}=0$ and transverse relaxation rate $R_{2,0}^{*}$. Under the static dephasing regime, the transverse relaxation rates of the paramagnetic and diamagnetic compartments are assumed to depend linearly on their respective susceptibility magnitudes: $R_{2,+,-}^{*}=a|\chi_{+,-}|+R_{2,0}^{*}$ [62], where $a=\frac{2\pi }{9\sqrt{3}}B_{0}$ and $B_{0}$ is the main magnetic field strength.

Owing to rapid T2^∗^ decay at 9.4 T, later echoes exhibit substantially reduced SNR; therefore, only the magnitude and QSM data from the first eight echoes (TE = 3.04–14.94 ms) were used as inputs to DECOMPOSE‐QSM for generating DCS and PCS maps.

### 2.5 Atlas Registration and Spatial Normalization

A mouse brain template containing 166 annotated brain regions [63, 64] was used to define cortical regions of interest (ROIs) for data analysis. ANTs [55] was used to register the mGRE magnitude image of each mouse brain to the template using symmetric normalization (SyN), including affine and deformable transformations. The resulting forward transformation fields were applied to warp the template labels to each individual mouse brain space, thereby obtaining subject‐specific ROIs. The corresponding inverse transformations were used to normalize individual QSM, DCS, and PCS maps to template space, enabling the generation of group‐averaged susceptibility maps for between‐model comparison.

### 2.6 Identification of Beta‐Amyloid Plaques on Susceptibility Maps

Plaque detection was performed using QSM‐PLAQUE Aβ detector [65], which identifies individual plaques by exploiting the low‐rank structure of local susceptibility data. The method uses orthogonal projection to separate structured background tissue signal from sparse, spatially distinct plaque signatures, followed by spatial‐spectral detection to localize individual plaques.

The detector was applied independently to the QSM and DCS maps to detect plaques within cortical regions. The detection performance obtained from the two maps was then compared quantitatively. To establish a reference standard, plaques were manually annotated on the central slice of each dataset by an experienced observer. The plaque detection results obtained from QSM and DCS were compared with the manual annotations using intersection over union (IoU), precision, recall, and F1 score.

The contrast‐to‐noise ratio (CNR) of each detected plaque was calculated using the surrounding 5 × 5 × 5‐voxel non‐plaque neighborhood as the reference. CNR was calculated using

(1) $$\text{CNR}=\frac{\left|{\bar{S}}_{\text{plaque}}-{\bar{S}}_{\text{surround}}\right|}{\sigma_{\text{surround}}}$$

where ${\bar{S}}_{\text{plaque}}$ denotes the mean susceptibility value within the detected plaque, and ${\bar{S}}_{\text{surround}}$ and $\sigma_{\text{surround}}$ denote the mean and standard deviation of the susceptibility values in the surrounding non‐plaque voxels, respectively.

### 2.7 Susceptibility Characterization of Beta‐Amyloid Plaques

For both the 5xFAD and $App^{\text{SAA}}$ datasets, plaques within cortical regions were detected as described above, and the mean QSM, DCS, and PCS values of the detected plaques were calculated. The relative diamagnetic contribution was quantified as the ratio of the absolute DCS magnitude to the total absolute susceptibility (|DCS|/(|DCS|+|PCS|)). Two‐sample t‐tests were performed to determine whether QSM and DCS values of detected plaques differed significantly (p<0.05) in the 5xFAD and $App^{\text{SAA}}$ datasets.

### 2.8 Effect of Spatial Resolution on Beta‐Amyloid Plaque Detection

High‐resolution imaging is essential for the detection and characterization of plaques because of the intrinsically small size of these lesions, which typically range from 10 to 50 μm in diameter [66]. Newly formed plaques have radii of only a few micrometers and gradually grow over time and can reach diameters of up to 200 μm at later disease stages through the clustering and merging of smaller plaques [67, 68]. To evaluate the influence of spatial resolution on plaque visibility and detection, we performed an image downsampling experiment on five mice (three 5xFAD and two $App^{\text{SAA}}$). The k‐space data, originally acquired at an isotropic resolution of 30 μm, were truncated in k‐space to simulate coarser isotropic resolutions of 45, 60, and 90 μm while maintaining a constant field of view. k‐space truncation was used to approximate lower‐resolution data acquisitions while retaining the noise characteristics of the original acquisition.

To assess the effect of voxel size on image quality, SNR was estimated from the magnitude image with TE = 9.84 ms at each spatial resolution. SNR was calculated from a central slice by dividing the mean signal intensity within the brain mask by the standard deviation of the background noise:

(2) $$\text{SNR}=\frac{{\bar{S}}_{\text{brain}}}{\sigma_{\text{background}}}$$

where ${\bar{S}}_{\text{brain}}$ is the mean signal intensity within the brain mask and $\sigma_{\text{background}}$ is the standard deviation of the noise measured in a signal‐free background region.

The full processing pipeline, including phase unwrapping, background field removal, QSM reconstruction, and DECOMPOSE‐QSM, was reapplied at each downsampled resolution. Plaques were detected at different resolutions using QSM‐PLAQUE Aβ detector, and plaque burden was quantified using plaque loading, defined as the percentage of each predefined ROI occupied by detected plaques:

(3) $$\text{Plaque Loading}=\frac{V_{\text{Plaques}}}{V_{\text{ROI}}}\cdot 100\%$$

where $V_{\text{Plaques}}$ is the total volume of detected plaques within the ROI and $V_{\text{ROI}}$ is the total volume of the ROI.

In addition, the effects of spatial resolution on susceptibility measurements were assessed at both the regional and plaque levels. At the regional level, the mean and standard deviation of QSM, DCS, and PCS values were calculated within five ROIs: S1BF, primary somatosensory cortex, barrel field; S1DZ, primary somatosensory cortex, dysgranular zone; M1, primary motor cortex; PIR, piriform cortex; and AMY, amygdala. To minimize susceptibility estimation errors, voxels containing prominent blood vessels or affected by artifacts in QSM were excluded using manually defined masks. For the plaque‐level analysis, connected components were identified using 6‐neighborhood connectivity to separate the detected plaque mask into individual plaques. Plaque‐size distributions were then characterized by plotting histograms of individual plaque volumes at each spatial resolution. The mean QSM, DCS, and PCS values within each detected plaque were also evaluated across resolutions to determine how spatial resolution affected plaque susceptibility quantification.

## 3 Results

### 3.1 Susceptibility Source Separation

**FIGURE 2:**
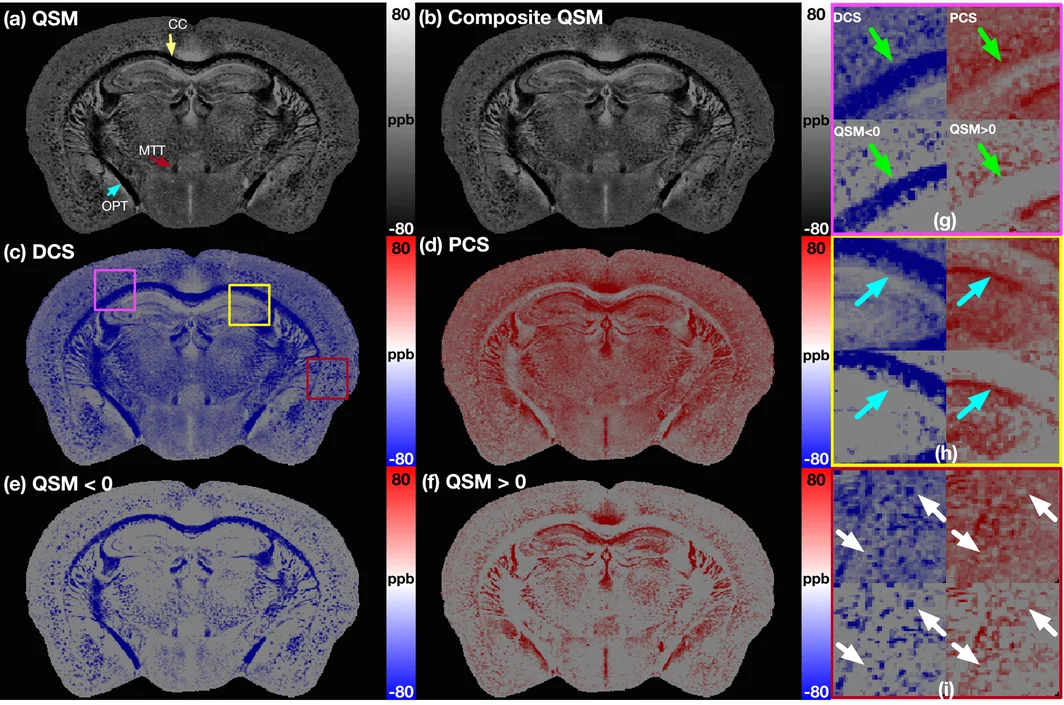
Ex vivo susceptibility maps from a representative 5xFAD mouse brain. (a) averaged QSM calculated from per‐echo QSM maps. Arrows indicate the corpus callosum (CC), optic tract (OPT), and mammillothalamic tract (MTT), three white‐matter structures with well‐characterized diamagnetic susceptibility. Scattered plaques in cortical and subcortical regions appear hypointense, suggesting predominantly diamagnetic susceptibility. (b) Composite QSM, calculated from DECOMPOSE‐QSM, showing close agreement with the average QSM in (a). (c, d) Diamagnetic component source (DCS) and paramagnetic component source (PCS) generated from DECOMPOSE‐QSM, revealing subvoxel mixtures of diamagnetic and paramagnetic contributions, which are not apparent in the thresholded QSM in (e, f). (e, f) Negative and positive component maps of QSM, obtained by thresholding the QSM map in (a) according to susceptibility sign. (g–i) zoomed‐in views of three representative regions, highlighting cortex and hippocampus (green and blue arrows) and individual plaques (white arrows), where DCS and PCS maps reveal the presence of subvoxel mixtures of paramagnetic and diamagnetic components that are not resolved in thresholded QSM.

Figure 2 shows the susceptibility maps of a representative 5xFAD mouse brain. The average QSM computed from per‐echo QSM maps (a) and the composite QSM generated using DECOMPOSE‐QSM (b) are in close agreement, confirming the consistency of the decomposition. The DCS (c) and PCS (d) maps revealed subvoxel mixtures of diamagnetic and paramagnetic components within both amyloid plaques and the corpus callosum, as indicated by the yellow arrows in QSM images. These features were not discernible in thresholded QSM images (e, f), demonstrating the added specificity of susceptibility decomposition over conventional QSM. Zoomed‐in views of three representative regions (g–i) further illustrated small plaques (white arrows) and local tissue structures (blue arrows). Aβ plaques appeared predominantly hypointense on QSM and DCS, suggesting that the diamagnetic contribution of aggregated amyloid outweighs the paramagnetic contribution. Qualitatively, DCS isolated the diamagnetic component with reduced contamination from paramagnetic sources, whereas PCS highlighted paramagnetic contributions.

**FIGURE A1:**
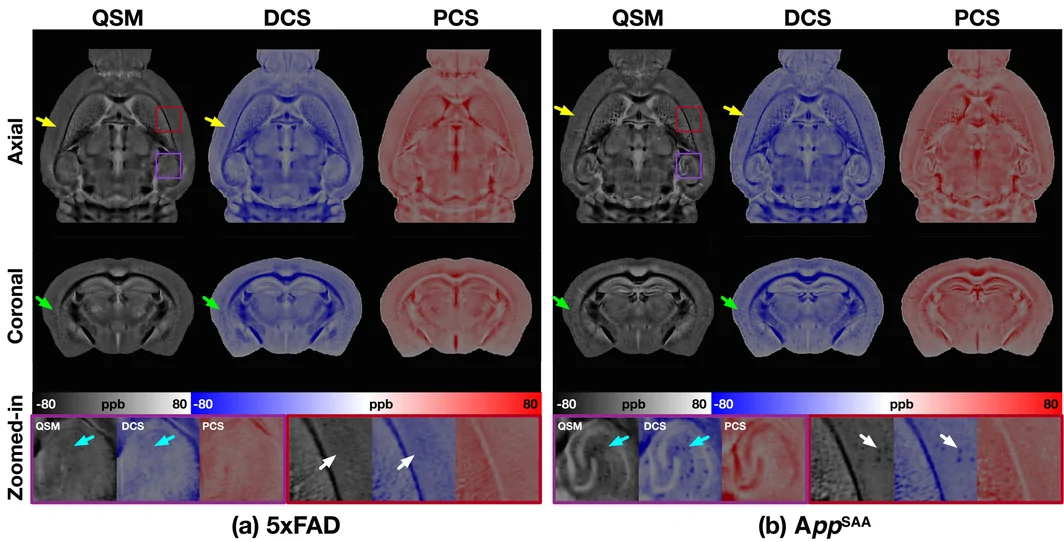
Group‐averaged QSM, DCS, and PCS maps of 5xFAD and $App^{\text{SAA}}$ mice in the common template space. The 5xFAD model exhibits dense plaque accumulation predominantly in the deeper cortical layers and subcortical regions (yellow and green arrows), whereas the $App^{\text{SAA}}$ model shows a more diffuse distribution of plaques across the cortex. Zoomed‐in views highlight the distinct spatial distribution patterns: 5xFAD displays clustered, dense plaque deposits (white arrows), whereas $App^{\text{SAA}}$ shows more widely dispersed plaques (blue arrows).

Group‐averaged susceptibility maps for 5xFAD and $App^{\text{SAA}}$ mice (Figure A1) revealed distinct spatial distribution patterns between the two models. The 5xFAD mice showed dense plaque accumulation predominantly in the deeper cortical layers and subcortical regions, whereas $App^{\text{SAA}}$ mice showed a more diffuse distribution of smaller plaques across the cortex, more closely resembling the spatial pattern of human amyloid pathology. These differences in plaque distribution motivated separate evaluation of detection performance and susceptibility characterization in each model, as described in the following sections.

### 3.2 Histological Data

**FIGURE 3:**
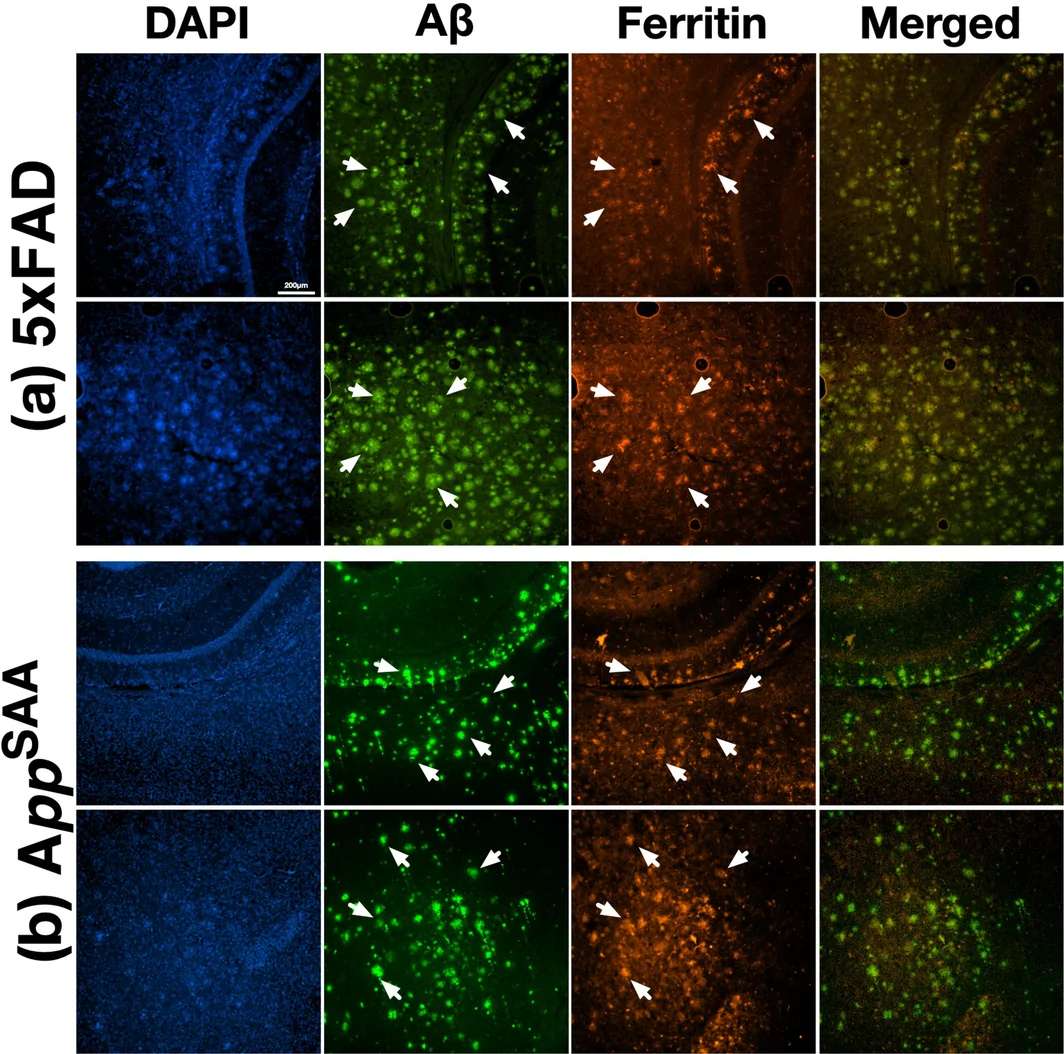
Immunofluorescence analysis of brain sections from 5xFAD (a) and $App^{\text{SAA}}$ (b) mice. From left to right: DAPI staining (blue) showing nuclear morphology; anti‐amyloid‐β immunostaining (Alexa Fluor 488, green), revealing amyloid‐β aggregates; anti‐ferritin immunostaining (Alexa Fluor 568, red), indicating ferritin deposits; and merged Aβ/ferritin staining overlay. The spatial overlap between Aβ and ferritin signals, indicated by white arrows, suggests that ferritin preferentially accumulates around amyloid aggregates.

**FIGURE A2:**
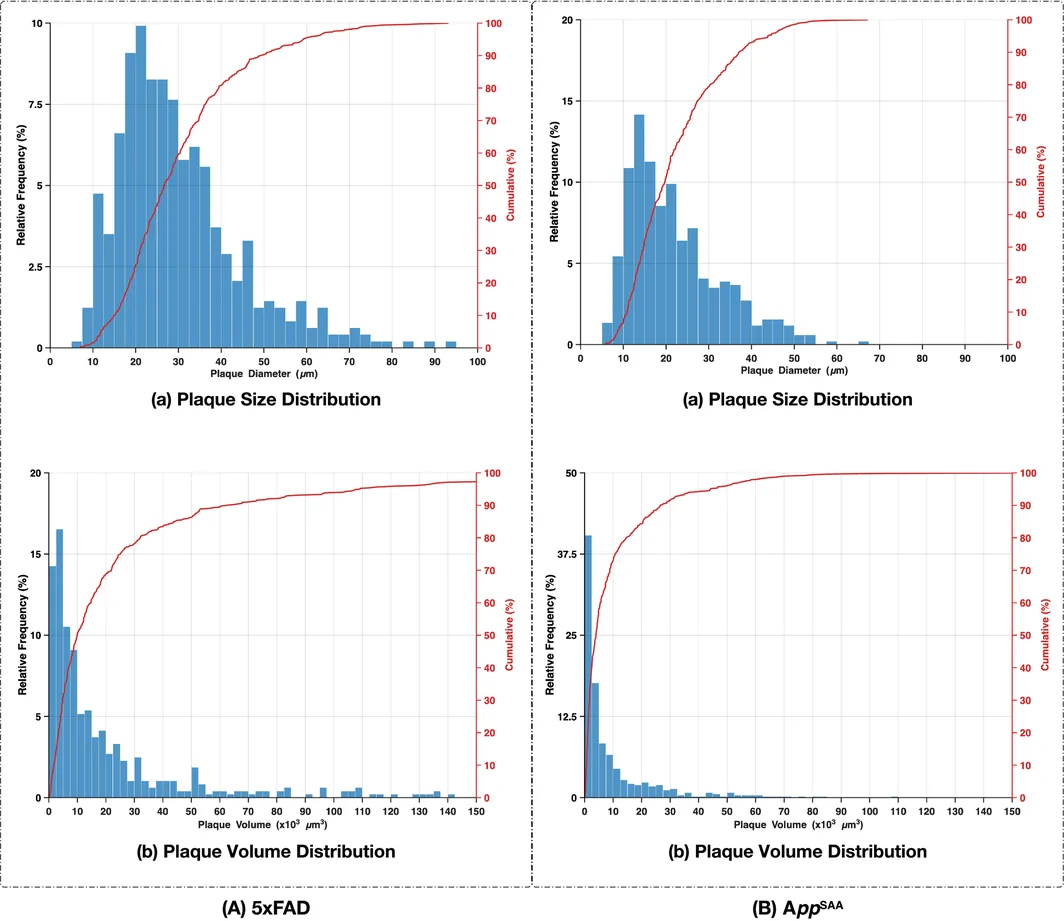
Histograms of plaque diameters and estimated volumes measured from Aβ‐stained histological images of 5xFAD (a) and $App^{\text{SAA}}$ (b) mice. In both models, most plaques had diameters less than 50 μm, although a subset of plaques in 5xFAD mice exceeded 70 μm. Plaque volumes were estimated by assuming a spherical geometry. The resulting volume distributions were right‐skewed, with most plaques having volumes below $6\times 10^{4}$ $\mu m^{3}$.

Figure 3 presents immunofluorescence staining of brain sections from 5xFAD and $App^{\text{SAA}}$ mice. DAPI staining demonstrated preserved tissue architecture and nuclear integrity in both models. Aβ immunostaining revealed abundant, densely distributed Aβ in 5xFAD mice, whereas $App^{\text{SAA}}$ exhibited a more scattered distribution of Aβ. Ferritin immunostaining identified ferritin‐positive regions, with notable enrichment in plaque areas in both models. The overlay of Aβ and ferritin staining showed marked spatial colocalization, as indicated by white arrows, supporting the presence of Aβ/ferritin aggregates in both 5xFAD and $App^{\text{SAA}}$ mice. Figure A2 shows the distribution of plaque diameters measured in Aβ‐stained histological images. Most plaques have diameters of less than 70 μm.

### 3.3 Identification of Beta‐Amyloid Plaques on Susceptibility Maps

**FIGURE 4:**
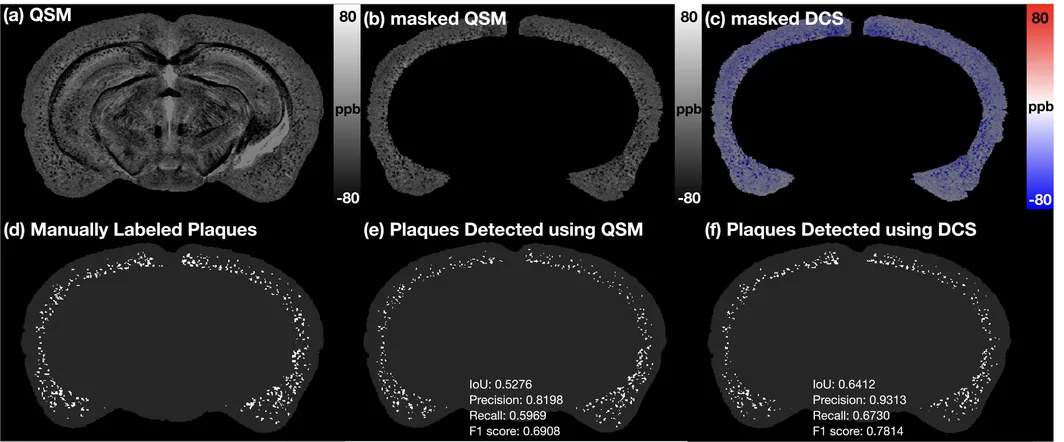
Comparison of plaque detection using QSM and DCS in a representative 5xFAD dataset. (a–c) QSM of the full brain, QSM and DCS masked to cortical regions, respectively. (d) Manually annotated plaques used as the reference standard. (e) Plaques detected on QSM. (f) Plaques detected on DCS. Detection performance was evaluated by comparing the results in (e) and (f) with the reference standard in (d) using intersection over union (IoU), precision, recall, and F1 score (values shown in each panel).

Figure 4 compares the plaque detection performance between QSM and DCS in a representative 5xFAD mouse. Plaques detected on QSM and DCS showed broadly similar spatial distributions and were generally consistent with the manually annotated reference standard. Quantitative evaluation showed that DCS outperformed QSM in terms of IoU, precision, recall, and F1 score. The mean ± standard deviation of plaque CNRs were 2.82±0.83 for QSM and 3.25±1.23 for DCS.

Quantitative evaluation of plaque detection performance is summarized in Table 1. In the 5xFAD mice, DCS achieved better IoU, precision, recall, and F1 scores than QSM. A similar pattern was observed in the $App^{\text{SAA}}$ mice, where DCS plaque detection yielded better quantitative scores than QSM. CNR analysis revealed higher plaque contrast in DCS than in QSM in both models: 3.04±0.27 vs. 2.74±0.22 in 5xFAD and 3.43±0.38 vs. 3.07±0.20 in $App^{\text{SAA}}$.

**TABLE 1:** Comparison of plaque detection performance between QSM and DCS in 5xFAD and $App^{\text{SAA}}$ mice at 30 μm isotropic resolution. Detection results were evaluated against a manually annotated reference standard using intersection over union (IoU), precision, recall, and F1 score. Values are reported as mean ± standard deviation.

Dataset	Map	IoU (↑)	Precision (↑)	Recall (↑)	F1 (↑)
5xFAD	QSM	0.60±0.03	0.83±0.03	0.69±0.06	0.75±0.03
	DCS	0.79±0.06	0.96±0.01	0.82±0.06	0.88±0.04
$App^{\text{SAA}}$	QSM	0.57±0.01	0.73±0.01	0.73±0.00	0.73±0.01
	DCS	0.77±0.05	0.88±0.05	0.86±0.01	0.87±0.03
Combined	QSM	0.59±0.03	0.79±0.06	0.70±0.05	0.74±0.02
	DCS	0.78±0.05	0.93±0.05	0.84±0.05	0.88±0.03

### 3.4 Effect of Spatial Resolution on Beta‐Amyloid Plaque Detection

**FIGURE 5:**
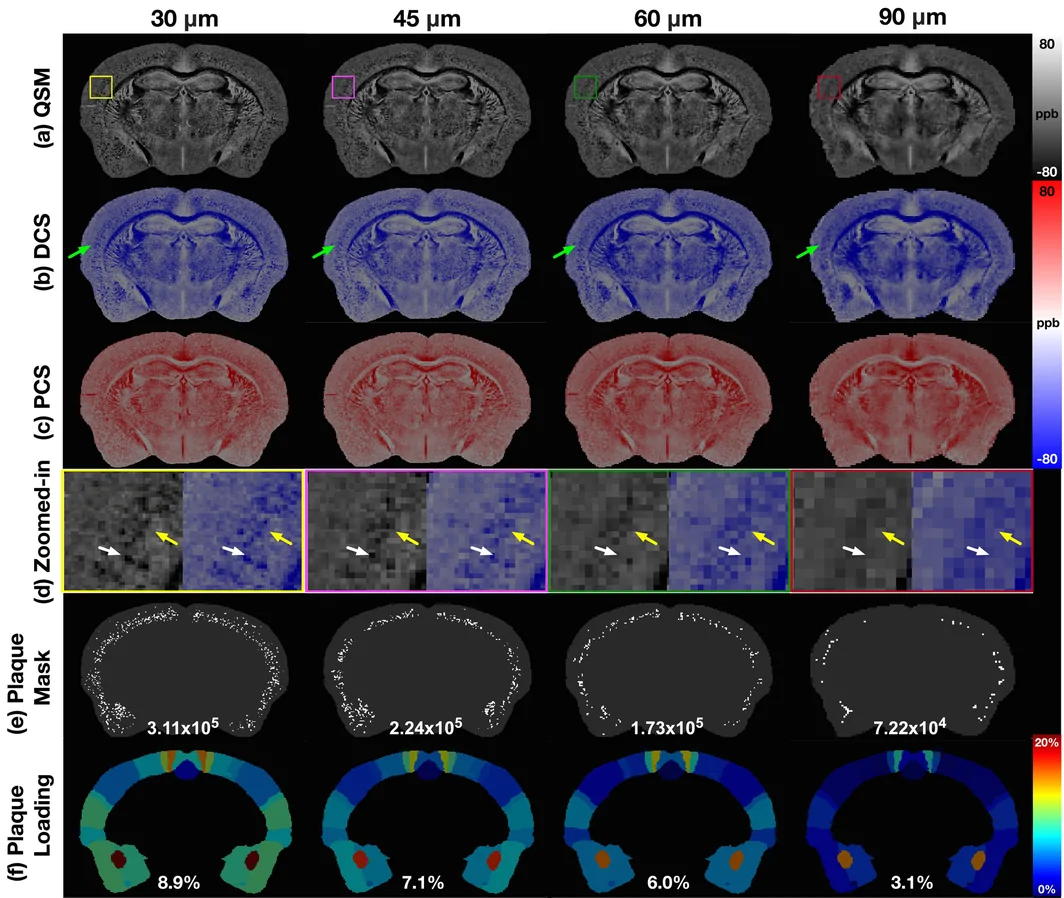
Effect of spatial resolution on susceptibility maps and plaque detection in a representative 5xFAD mouse. The data were acquired at an isotropic resolution of 30 μm and subsequently downsampled to isotropic resolutions of 45, 60, and 90 μm. As the spatial resolution becomes coarser, fine structural features become increasingly blurred and difficult to discern. In the zoomed‐in views (d), both small and large plaques (yellow and white arrows) progressively lose definition and become difficult to identify at resolutions of 60 μm and 90 μm. (e) plaques detected within the cortex at each resolution, demonstrating a reduction in the number of detectable plaques as spatial resolution becomes coarser. The total volumes of the detected plaques are reported in the figure, in units of μm^3^, showing a progressive decrease in detected plaque volume. (f) plaque loading computed from the detected plaques within predefined cortical ROIs, showing a consistent decline with coarser spatial resolution. Whole‐cortex plaque loading values were 8.9*%*, 7.1*%*, 6.0*%*, and 3.1*%* at resolutions of 30, 45, 60, and 90 μm, respectively.

Figure 5 shows QSM (a), DCS (b), and PCS (c) maps from a representative 5xFAD mouse at isotropic spatial resolutions of 30, 45, 60, and 90 μm. As spatial resolution became coarser, fine structural features, particularly amyloid plaques, became increasingly blurred and less discernible. In the zoomed‐in views (d), both small and large plaques were clearly distinguishable at 30 and 45 μm, but became progressively blurred at coarser spatial resolutions. At 90 μm, small plaques were no longer visible, whereas larger plaques remained detectable at moderate resolutions but gradually lost definition and became difficult to delineate at 60 and 90 μm. The plaque detection results (e) showed a reduction in both the number and volume of detectable plaques as spatial resolution became coarser. Correspondingly, plaque loading (f) also decreased at coarser resolutions. The plaque loading in the whole cortical region declined from 8.9% at 30 μm to 7.1%, 6.0%, and 3.1% at 45, 60, and 90 μm, respectively. The calculated SNR values were 14.7, 19.8, 35.0, and 65.4 at 30, 45, 60, and 90 μm isotropic resolutions, respectively, confirming the expected increase in SNR with increasing voxel volume.

**FIGURE 6:**
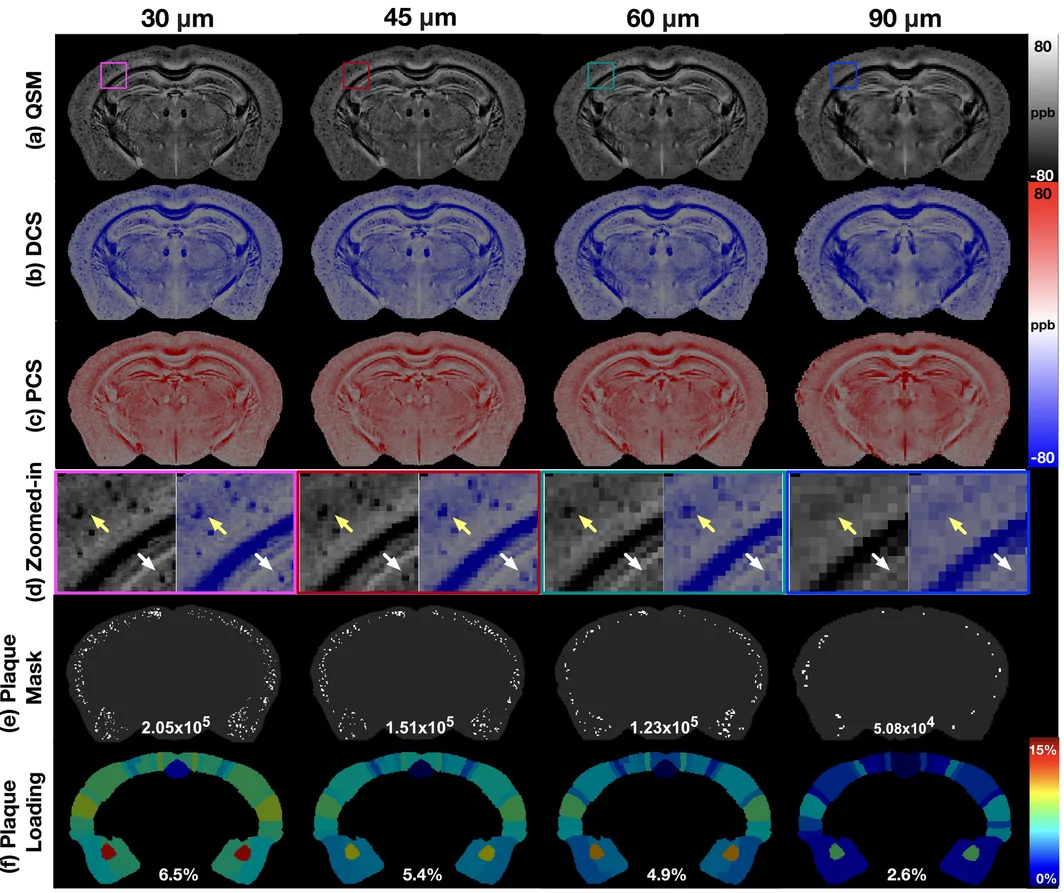
Effect of spatial resolution on susceptibility maps and plaque detection in a representative $App^{\text{SAA}}$ mouse. The data were acquired at an isotropic resolution of 30 μm and subsequently downsampled to isotropic resolutions of 45, 60, and 90 μm. (a–c) QSM, DCS, and PCS maps at each spatial resolution. (d) zoomed‐in views showing the progressive loss of plaque visibility (yellow and white arrows), with increasingly coarse resolution. At 90 μm, most plaques are no longer identifiable. (e) plaques detected at each resolution, demonstrating a reduction in the number of detectable plaques as spatial resolution becomes coarser. The total detected plaque volume decreased from $2.05\times 10^{5}$ μm^3^ at 30 μm to $1.51\times 10^{5},\;1.23\times 10^{5}$, and $5.08\times 10^{4}$ μm^3^ at 45, 60, and 90 μm, respectively. (f) plaque loading computed from detected plaques within predefined cortical ROIs, showing a consistent decline as spatial resolution becomes coarser. Whole‐cortex plaque loading values were 6.5*%*, 5.4*%*, 4.9*%*, and 2.6*%* at resolutions of 30, 45, 60, and 90 μm, respectively.

Figure 6 presents susceptibility maps, plaque detection results, and plaque loading measurements at multiple spatial resolutions in a representative $App^{\text{SAA}}$ mouse. Consistent with the observations in Figure 5, coarser spatial resolution leads to a progressive blurring of amyloid plaques (yellow and white arrows in (d)), making them increasingly difficult to discern. Consequently, both the number and total volume of detected plaques decrease, resulting in lower plaque loading values at coarser resolutions. SNR values were 15.6, 22.2, 36.4, and 65.6 at isotropic resolutions of 30, 45, 60, and 90 μm, respectively.

**FIGURE 7:**
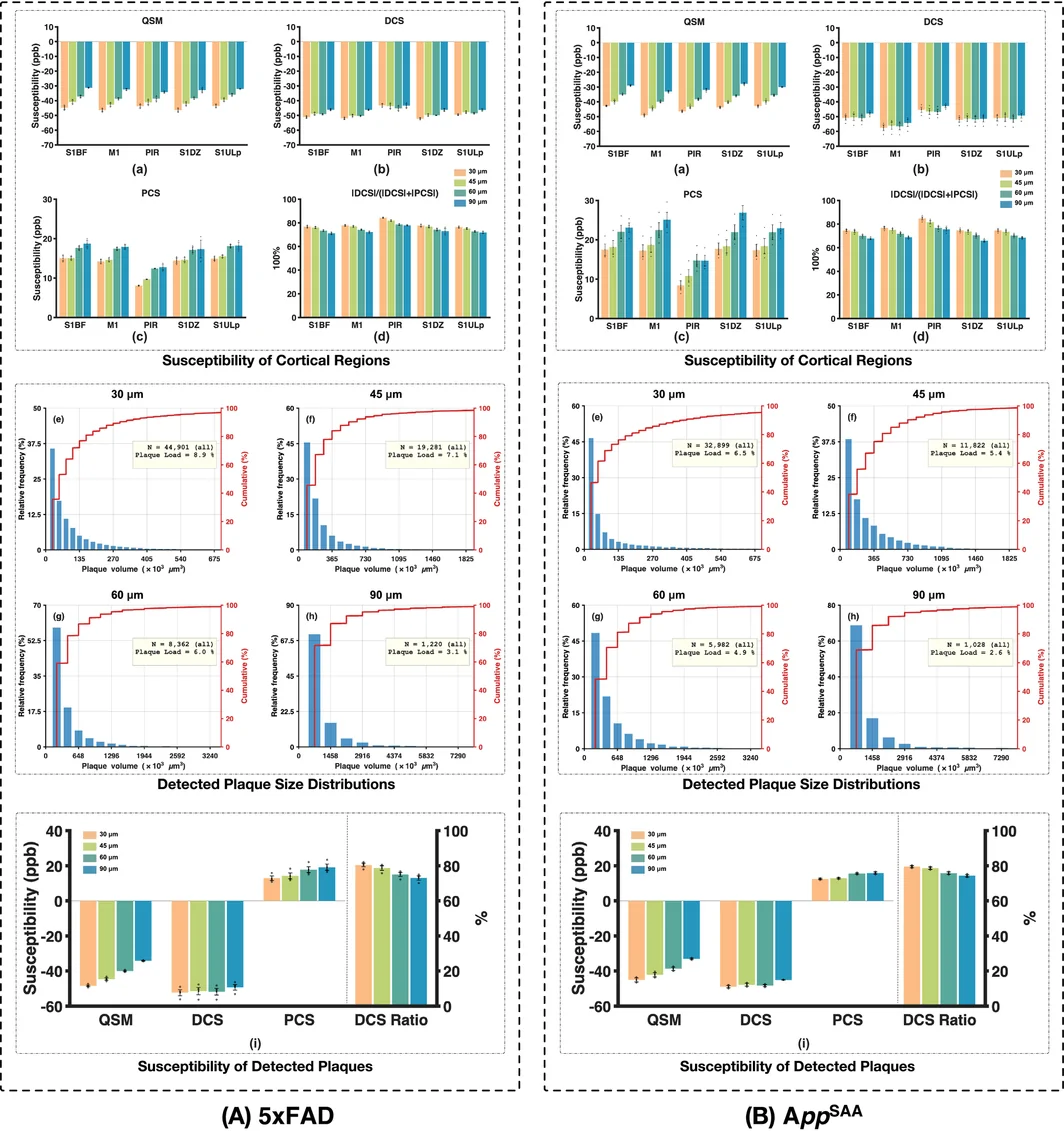
Effect of spatial resolution on regional susceptibility, plaque size distribution, and plaque susceptibility in 5xFAD (A) and $App^{\text{SAA}}$ (B) mice. Data were acquired at an isotropic resolution of 30 μm and downsampled to isotropic resolutions of 45, 60, and 90 μm. (a–c) Mean QSM, DCS, and PCS values across five cortical regions at each resolution. The magnitudes of the QSM and DCS values decrease as spatial resolution becomes coarser, whereas PCS values increase. (d) Relative DCS contribution, defined as |DCS|/(|DCS|+|PCS|), across cortical regions and resolutions. (e–h) Histograms of detected plaque volumes at 30, 45, 60, and 90 μm from a representative 5xFAD mouse and an $App^{\text{SAA}}$ mouse, showing relative frequency (blue bars, left axis; normalized by total plaque count) and cumulative distribution (red line, right axis). In the 5xFAD mouse, the total plaque count decreased from 44,901 at 30 μm to 1220 at 90 μm, with plaque loading decreasing from 8.9% to 3.1%. A similar pattern was observed in the $App^{\text{SAA}}$ mouse, in which the plaque count decreased from 32,899 to 1028 and plaque loading decreased from 6.5% to 2.6%. (i) Mean QSM, DCS, PCS, and DCS ratio of individual detected plaques at each resolution from 5xFAD and $App^{\text{SAA}}$ mice. As spatial resolution becomes coarser, QSM and PCS values shift toward more paramagnetic values, whereas the relative DCS contribution decreases. (S1BF: primary somatosensory cortex, barrel field; M1: primary motor cortex; PIR: piriform cortex; S1DZ: primary somatosensory cortex, dysgranular zone; S1ULp: primary somatosensory cortex, upper lip region.).

Figure 7 presents a detailed analysis of the resolution‐dependent effects on susceptibility and plaque detection in 5xFAD (A) and $App^{\text{SAA}}$ (B) mice. Regional susceptibility analysis across five cortical regions (a–d) revealed that QSM and DCS values decreased in magnitude as spatial resolution became coarser, whereas PCS values increased. The relative DCS contribution, defined as |DCS|/(|DCS|+|PCS|), decreased with coarser image resolution. Plaque detection results from representative 5xFAD and $App^{\text{SAA}}$ mice (e–h) indicated that small plaques predominated at all resolutions. As spatial resolution became coarser, both the number of detected plaques and the estimated plaque loading decreased. In the 5xFAD mouse, the plaque count decreased from 44,901 at 30 μm to 1220 at 90 μm, accompanied by a reduction in plaque loading from 8.9% to 3.1%. Similarly, in the $App^{\text{SAA}}$ mouse, the plaque count decreased from 32,899 to 1028, whereas plaque loading declined from 6.5% to 2.6% over the same resolution range. At plaque level (i), coarser spatial resolution shifted the QSM and PCS values toward more paramagnetic values while reducing the relative DCS contribution.

### 3.5 Susceptibility Characterization of Beta‐Amyloid Plaques

**FIGURE 8:**
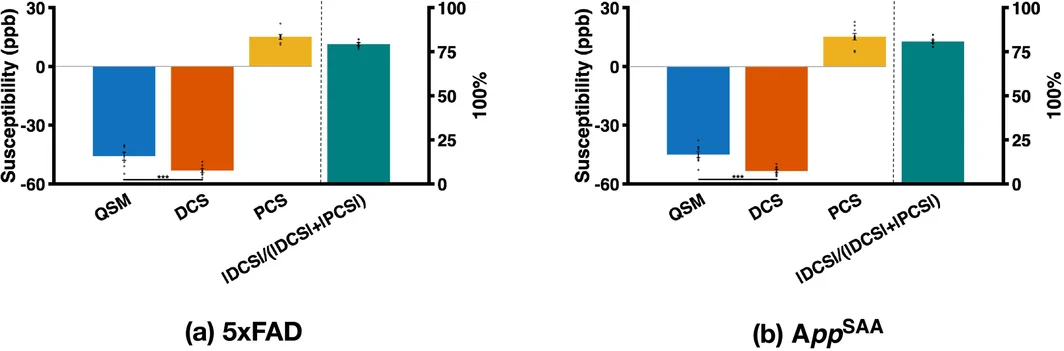
Plaque susceptibility characterization in cortical regions of 5xFAD (a) and $App^{\text{SAA}}$ (b) mice. Mean QSM, DCS, PCS, and relative DCS contribution (|DCS|/(|DCS|+|PCS|)) were calculated for detected plaques in each mouse. The high DCS contribution values indicate that diamagnetic susceptibility is the dominant component within plaques in both models. Statistically significant differences between QSM and DCS values were observed in both 5xFAD and $App^{\text{SAA}}$ (two‐sample t‐test, 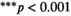).

Figure 8 summarizes the plaque‐level measurements of QSM, DCS, PCS, and DCS contribution in the 5xFAD and $App^{\text{SAA}}$ mice. In both models, the detected plaques exhibited predominantly diamagnetic susceptibility, with the DCS contribution being approximately 80*%*. Two‐sample t‐tests showed significant differences between QSM and DCS values in both models.

## 4 Discussion

In this study, we acquired and analyzed ex vivo high‐resolution mGRE data from 5xFAD and $App^{\text{SAA}}$ mouse models of AD. From these data, QSM and paramagnetic and diamagnetic susceptibility components were reconstructed to characterize the magnetic susceptibility properties of amyloid plaques. Our main findings were as follows: (1) DCS and PCS provided complementary information about diamagnetic and paramagnetic plaque‐associated susceptibility sources that could not be readily resolved using QSM alone; (2) DCS enabled more reliable plaque detection than QSM and provided higher plaque‐level CNR; (3) detected plaques in both 5xFAD and $App^{\text{SAA}}$ mice were predominantly diamagnetic but also exhibited a paramagnetic contribution associated with plaque‐related iron accumulation, as supported by immunofluorescence histology; and (4) high spatial resolution was critical for plaque visualization, detection, and susceptibility quantification.

### 4.1 Identification of Beta‐Amyloid Plaques

As shown in Figures 2 and 4, Aβ plaques were clearly visualized on both QSM and DCS. The detected plaques exhibited higher CNR values on DCS than on QSM, which may explain the improved plaque detection performance observed with DCS. This difference may be attributed to the predominantly diamagnetic susceptibility of the plaques, which produces stronger contrast on DCS. In contrast, QSM reflects the net magnetic susceptibility arising from both diamagnetic and paramagnetic sources. Consequently, the visibility of diamagnetic plaques on QSM may be reduced by coexisting paramagnetic substances.

Several factors may influence the comparison of plaque detection performance between QSM and DCS. The reference standard was established using manual annotation, which may introduce observer‐dependent variability. Detection performance may also depend on the selected algorithm and its hyperparameters. Despite these limitations, the plaque detection results obtained from QSM and DCS were broadly consistent. Future studies involving larger cohorts, multiple observers, and independent detection methods are needed to determine whether DCS consistently outperforms QSM for plaque identification.

Histological techniques are essential for visualizing amyloid plaques in AD because of their high resolution [69, 70, 71]. Stains such as Congo Red and Thioflavin S bind specifically to Aβ fibrils [72, 73], whereas immunohistochemistry (IHC) uses antibodies against Aβ peptides [74] for plaque detection. Advanced imaging methods such as confocal and multiphoton microscopy enable three‐dimensional visualization and analysis of plaque‐cell interactions. However, histology is prone to variability introduced during tissue processing and sectioning, and is inherently limited to sampled regions rather than providing whole‐brain coverage. Traditional dyes detect mainly mature plaques, and quantification can be subjective. In addition, histology cannot assess functional changes, requiring complementary imaging techniques, such as PET or MRI, to provide a more comprehensive characterization of AD pathology [75]. In this study, we showed that QSM and susceptibility source separation provide quantitative information about tissue magnetic susceptibility and facilitate a more comprehensive investigation of AD‐related pathology.

### 4.2 Effect of Spatial Resolution on Beta‐Amyloid Plaque Detection

High spatial resolution is critical for reliable visualization, detection, and susceptibility quantification of amyloid plaques. The downsampling experiments (Figures 5 and 6) demonstrated that partial volume effects became increasingly pronounced as spatial resolution decreased, resulting in the progressive blurring of plaque boundaries and the complete loss of detectability for small plaques. This degradation directly affected plaque detection, resulting in fewer detected plaques and lower plaque burden measurements.

Conventional in vivo QSM studies in human subjects are typically limited to resolutions on the order of ∼1 mm because of hardware constraints, motion artifacts, acquisition time limitations, and SNR considerations [76]. Human Aβ plaques range from approximately 10 μm to more than 200 μm in diameter, depending on plaque subtype, disease stage, and brain region [77]. At conventional in vivo resolutions, individual plaques therefore fall below the spatial resolution limit and cannot be distinguished from the surrounding tissue. Consequently, susceptibility analyses are restricted to regional changes. In vivo murine imaging faces similar challenges. Although plaque visualization has been demonstrated in living mice [78], image quality is substantially degraded by physiological motion, and the achievable resolution remains insufficient for reliable plaque susceptibility characterization. In contrast, our ex vivo approach, with an isotropic resolution of 30 μm, enabled direct visualization of individual plaques and assessment of their susceptibility properties. This provides a level of microstructural detail that is not currently achievable with in vivo imaging protocols. However, such high spatial resolution inherently reduces SNR because of the smaller voxel volume, requiring careful optimization of acquisition parameters to balance spatial resolution, SNR, and scan time. Advances in ultra‐high‐field systems, accelerated acquisition strategies, and deep learning‐based methods may help narrow the resolution gap between ex vivo and in vivo imaging.

The downsampling experiments demonstrated that high spatial resolution is essential for accurate susceptibility quantification. This observation is consistent with previous work. Zhou et al. [79] demonstrated systematic underestimation of absolute QSM values with increasing voxel size. Karsa et al. [80] reported that reduced spatial resolution decreases image contrast and increases systematic errors in QSM. Our resolution analysis (Figure 7) showed that the magnitudes of the QSM and DCS values decreased at both the regional and plaque levels as spatial resolution decreased, whereas PCS values exhibited an increasingly paramagnetic trend. This behavior likely reflects a combination of partial volume averaging and insufficient sampling of susceptibility‐induced field perturbations at coarser resolutions. In addition, the susceptibility decomposition algorithm itself may influence the accuracy of diamagnetic and paramagnetic estimates at different spatial resolutions. A systematic investigation of the resolution dependence of susceptibility decomposition methods warrants further study.

The histograms of detected plaque volumes (Figure 7) revealed strongly right‐skewed distributions in both mouse models. Most detected plaques fell within the smallest volume bins and progressively fewer plaques were at larger‐volume bins. This size distribution is consistent with prior histological studies reporting that plaque populations in AD mouse models are dominated by small deposits [67, 68]. Histological measurements (Figure A2) showed that plaque diameters in both models were predominantly below 70 μm and the distributions of estimated plaque volume were right‐skewed. This finding is consistent with the right‐skewed plaque size distributions observed in the MRI‐based detection analysis. The predominance of small plaques has important implications for imaging and detection. MRI data in this study were acquired at an isotropic resolution of 30 μm. Plaques substantially smaller than the voxel dimensions are therefore unlikely to be individually resolved, whereas closely spaced plaques may have appeared as a single larger plaque because of partial volume effects. Consistent with this possibility, plaque‐volume histograms obtained from the 30μm MRI data showed that approximately 25% of the detected plaques in both models had measured volumes exceeding $1.35\times 10^{5}\mu m^{3}$, whereas the histology‐derived estimates indicated that most plaques had volumes below $1.0\times 10^{5}\mu m^{3}$. The histological analysis was based on 2D measurements from a limited number of image fields and therefore did not capture the full 3D extent or complete size distribution of the plaques. Although MRI‐ and histology‐derived plaque volumes are not directly comparable, this discrepancy suggests that the limited spatial resolution of MRI, together with partial volume effects, may lead to overestimation of plaque size and the merging of adjacent plaques into single detected objects. Because most plaques are intrinsically small, they are particularly susceptible to partial volume effects and become increasingly difficult to detect as spatial resolution becomes coarser. This effect explains the rapid decline in plaque counts and estimated plaque burden observed in the downsampling experiments. The skewed size distribution suggests that conventional low‐resolution imaging studies may systematically underrepresent the true plaque population by detecting primarily the largest plaques while missing the more numerous small deposits. This detection bias may also influence susceptibility characterization. At coarser resolutions, the detected plaque population becomes increasingly weighted toward larger plaques, which may exhibit different relative diamagnetic and paramagnetic contributions. This shift may partly explain the resolution‐dependent changes observed in the QSM, DCS, and PCS measurements.

### 4.3 Susceptibility Characterization of Beta‐Amyloid Plaques

The detected plaques in both 5xFAD and $App^{\text{SAA}}$ mice exhibited predominantly diamagnetic susceptibility accompanied by an additional paramagnetic component (Figure 8). This finding is consistent with previous studies showing that amyloid plaques contain both diamagnetic amyloid‐beta aggregates and paramagnetic iron deposits. Iron accumulation in the AD brain is a well‐established pathological feature that contributes to neurodegeneration through mechanisms such as oxidative stress, ferroptosis, and enhanced protein aggregation [81]. The histological findings in the present study support the coexistence of amyloid and iron within plaques. Immunofluorescence staining demonstrated the colocalization of Aβ aggregates and ferritin deposits in both 5xFAD and $App^{\text{SAA}}$ mice (Figure 3), supporting the presence of diamagnetic and paramagnetic sources within plaques. This observation is consistent with substantial evidence that iron accumulation is a prominent feature of AD pathology [25, 26] and provides biological support for the susceptibility patterns observed on DCS and PCS maps.

The coexistence of diamagnetic sources, such as Aβ, and paramagnetic sources, such as iron, within plaques presents a fundamental challenge for conventional QSM. Although aggregated amyloid protein is intrinsically diamagnetic, iron accumulation within plaques introduces a strong paramagnetic contribution that can partially offset the diamagnetic signal. Consequently, QSM may not accurately reflect the individual contributions of amyloid and iron, and plaques with substantial iron accumulation may even exhibit net paramagnetic susceptibility. Indeed, several studies have reported plaques with positive susceptibility values [25, 26]. Susceptibility‐source separation reduces the ambiguity caused by cancellation between opposing susceptibility sources and therefore enables a more specific characterization of plaque‐associated magnetic components than QSM alone.

Our findings extend the previous work of Gong et al. [17], who demonstrated that susceptibility mapping can detect amyloid plaque accumulation in mouse models of AD and showed that diamagnetic and paramagnetic susceptibility maps provide complementary contrast related to amyloid and iron deposition. However, their study primarily focused on plaque visualization and histological validation and did not perform plaque detection or detailed characterization of plaque susceptibility components. By combining plaque detection with susceptibility decomposition, our study provides a more comprehensive assessment of susceptibility composition at the plaque level. The present results also underscore the importance of high‐resolution imaging for plaque characterization. As demonstrated by our resolution analysis, susceptibility estimates become increasingly biased at lower spatial resolutions because of partial volume effects and insufficient sampling of plaque‐induced field perturbations.

### 4.4 Limitations and Future Directions

Several limitations of this study should be acknowledged and addressed in future investigations. First, the biological specificity of susceptibility decomposition remains limited. DCS and PCS are not unique biomarkers of amyloid and iron, respectively; rather, they reflect the combined contributions of all diamagnetic and paramagnetic susceptibility sources within a voxel. Although Aβ plaques were predominantly diamagnetic in our study, the observed DCS signal cannot be attributed exclusively to amyloid aggregates. Similarly, PCS reflects all paramagnetic contributions, including iron, deoxyhemoglobin, inflammatory processes, and other tissue components that alter magnetic susceptibility. Second, the present study employed the DECOMPOSE‐QSM framework, which is based on $R_{2}^{*}$ measurements without incorporating $R_{2}^{\prime }$ measurements. As noted by Ji et al. [34] and Oliveira et al. [35], $R_{2}^{*}$‐based susceptibility separation methods may be less accurate than $R_{2}^{\prime }$‐based methods. Consequently, the estimated DCS and PCS maps may be affected by methodological biases specific to the reconstruction framework. Comparison among different susceptibility separation methods in this context warrants further investigation. Third, despite the high spatial resolution achieved in this study, imaging resolution remained a limiting factor. Even at an isotropic resolution of 30 μm, some small plaques remained below the detection threshold, and partial volume effects may influence susceptibility values. As demonstrated by our resolution analysis, decreasing spatial resolution leads to attenuation of plaque susceptibility contrast and loss of plaque detectability. Further improvements in spatial resolution would enable a more complete characterization of plaque populations. Fourth, all imaging was performed ex vivo on perfusion‐fixed tissue at 9.4 T, which enables substantially higher spatial resolution and SNR than in vivo imaging. However, the perfusion and fixation alter tissue properties relative to the in vivo state and affect the appearance of vascular structures, atherosclerotic plaques, and their magnetic susceptibility measurements [82]. Formalin fixation can alter tissue magnetic susceptibility and render tissue less diamagnetic than fresh tissue, an effect attributed to the paramagnetic contributions of formaldehyde and protein cross‐linking rather than changes in water content [82, 83]. Although the relative susceptibility contrast between gray and white matter is broadly preserved under ex vivo conditions, the absence of blood flow eliminates the paramagnetic contribution of deoxyhemoglobin, altering vascular susceptibility contrast [84]. These fixation‐related changes must be considered when relating ex vivo findings to in vivo conditions, and translation to in vivo studies will require further investigation. Translating plaque‐level susceptibility analysis to preclinical and clinical in vivo imaging remains challenging because the achievable resolution is constrained by physiological motion, scan time, and limited SNR. Further technical development will therefore be required to translate the present approach to in vivo applications.

## 5 Conclusion

This study demonstrates the value of combining high‐resolution QSM with susceptibility separation to characterize amyloid plaque pathology in the 5xFAD and $App^{\text{SAA}}$ mouse models of AD. Our results show that DCS and PCS provide complementary information about plaque composition that cannot be readily resolved using QSM alone. In particular, DCS provided improved plaque contrast and enabled more reliable detection of individual Aβ plaques. Systematic downsampling experiments further demonstrated that spatial resolution is a critical determinant of plaque detectability and susceptibility quantification. Histological data showed spatial overlap between Aβ aggregates and ferritin accumulation, supporting the coexistence of diamagnetic and paramagnetic contributions within plaques observed on DCS and PCS maps. Together, these findings establish high‐resolution susceptibility mapping and source separation as promising MRI‐based tools for assessing plaque accumulation, characterizing plaque‐associated magnetic properties, and supporting preclinical investigation of AD pathology.

## Author Contributions

**Juan Liu:** conceptualization, data curation, formal analysis, investigation, methodology, software, validation, visualization, writing – original draft; writing – review and editing. **Jingjia Chen:** conceptualization, methodology, supervision, validation, writing – review and editing. **Season K. Wyatt‐Johnson:** methodology, resources. **Jie Chen:** methodology, resources. **Zhuoheng Liu:** methodology, resources. **Hongbo Wu:** methodology, resources. **Li Feng:** methodology, resources. **Randy R. Brutkiewicz:** methodology, resources. **Nian Wang:** conceptualization, funding acquisition, methodology, project administration, resources, software, supervision, validation, writing – original draft, writing – review and editing.

## Funding

This work was supported by the National Institutes of Health Grant, United States, Grant/Award Number: R01 NS125020; Ralph W. and Grace M. Showalter Research Award; the UT Rising STARs award.

## Conflicts of Interest

The authors declare no conflicts of interest.
